# Supplementary material for: Comparative study of artificial light plant factories and greenhouse seedlings of SAOPOLO tomato
Source: PLoS One. 2025 Mar 5;20(3):e0314808. doi: 10.1371/journal.pone.0314808 (PMC11882041; doi:10.1371/journal.pone.0314808)
Supplement: S1 Table — (DOC) [file pone.0314808.s001.doc]

# Comparative study of artificial light plant factories and greenhouse seedlings of SAOPOLO tomato

Jun Zou*，Wenbin Liu ，Dawei Wang，Shipeng Luo， Shaojun Yang，Xiaotao Ding Mingming Shi*

1 School of Science, Shanghai Institute of Technology, No.100, Haiquan Road, Fengxian District, Shanghai 201418, China;

2 Shanghai Youyou Agricultural Technology Co., Ltd., Shanghai 202150, China

3 Shanghai Academy of Agricultural Sciences, Shanghai 201403, China

* Correspondence: zoujun@sit.edu.cn (J.Z.); mmshi@sit.edu.cn

# S1 Table

**S1.1 Figure 6 30-day environmental monitoring data for plant factories**

| Time（day） | Environmental temperature（℃） | Environmental humidity（%） | Ambient carbon dioxide concentration（ppm） |
| --- | --- | --- | --- |
| 1 | 25.61 | 85.38 | 481.72 |
| 2 | 24.8 | 87.47 | 486.42 |
| 3 | 24.15 | 85.1 | 479.14 |
| 4 | 24.97 | 86.51 | 471.55 |
| 5 | 25.09 | 86.31 | 450.06 |
| 6 | 24.95 | 89.75 | 486.19 |
| 7 | 24.63 | 89.47 | 495.98 |
| 8 | 25.91 | 85.15 | 464.08 |
| 9 | 24.22 | 86.13 | 497.45 |
| 10 | 25 | 85.51 | 472.23 |
| 11 | 25.11 | 85.64 | 499.54 |
| 12 | 24.57 | 87.6 | 460.9 |
| 13 | 25.2 | 89.02 | 456.85 |
| 14 | 25.85 | 89.61 | 458.72 |
| 15 | 25.81 | 88.49 | 493.77 |
| 16 | 25.96 | 89.05 | 463.99 |
| 17 | 24.86 | 89.62 | 476.45 |
| 18 | 24.64 | 88.62 | 478.62 |
| 19 | 25.89 | 89.61 | 468.14 |
| 20 | 24.41 | 85.12 | 462.22 |
| 21 | 24.66 | 88.78 | 473.9 |
| 22 | 25.73 | 87.02 | 470.9 |
| 23 | 25.56 | 85.03 | 456.89 |
| 24 | 25.33 | 89.58 | 453.71 |
| 25 | 25.48 | 89.36 | 456.49 |
| 26 | 24.63 | 85.97 | 492.69 |
| 27 | 25.9 | 86.92 | 467.75 |
| 28 | 25.67 | 85.92 | 455.95 |
| 29 | 25.24 | 85.61 | 457.47 |
| 30 | 24.48 | 85.31 | 456.69 |

**S1.2 Figure 7 Leaf length (Fig. A,B,C) and leaf width (Fig. D,E,F) of the 1st-3rd true leaves of the two nursery methods in plant factories and greenhouses**

2.1 The table shows the five sets of data and their means and standard deviations in Figure A（Inside）.

| Time（day） | data 1（cm） | data 2（cm） | data 3（cm） | data 4（cm） | data 5（cm） | average（cm） | standard deviation |
| --- | --- | --- | --- | --- | --- | --- | --- |
| 6 | 1.8 | 2.8 | 2.6 | 2.4 | 2.7 | 2.3 | 0.36 |
| 7 | 6 | 7.6 | 7.3 | 6.3 | 7.2 | 6.8 | 0.62 |
| 8 | 11.6 | 13 | 12.7 | 11.9 | 12.7 | 12.3 | 0.53 |
| 9 | 19.8 | 22.8 | 22.2 | 20.4 | 21.7 | 21.3 | 1.12 |
| 10 | 38.8 | 40.2 | 39.9 | 39.1 | 39.9 | 39.5 | 0.53 |
| 11 | 43.6 | 46.4 | 45.9 | 44.1 | 45.4 | 45. | 1.06 |
| 12 | 47.1 | 55.7 | 54 | 48.8 | 51.8 | 51.4 | 3.18 |
| 13 | 55.6 | 61.8 | 60.6 | 56.8 | 59.1 | 58.7 | 2.31 |
| 14 | 59.3 | 64.7 | 63.7 | 60.3 | 62.4 | 62.0 | 2.02 |
| 15 | 69.7 | 76.3 | 75 | 71 | 73.4 | 73.0 | 2.45 |
| 16 | 76.8 | 78.6 | 78.3 | 77.1 | 78.1 | 77.7 | 0.7 |
| 17 | 75.3 | 84.3 | 82.5 | 77.1 | 80.2 | 79.8 | 3.32 |
| 18 | 76.6 | 85 | 83.4 | 78.2 | 81.2 | 80.8 | 3.13 |
| 19 | 82.3 | 91.7 | 89.9 | 84.1 | 87.4 | 87.0 | 3.5 |

2.2 The table shows the five sets of data and their means and standard deviations in Figure A（Outside）.

| Time（day） | data 1（cm） | data 2（cm） | data 3（cm） | data 4（cm） | data 5（cm） | average（cm） | standard deviation |
| --- | --- | --- | --- | --- | --- | --- | --- |
| 7 | 7 | 9.2 | 8.8 | 7.5 | 8.2 | 8.1 | 0.84 |
| 8 | 11.7 | 14.4 | 13.9 | 12.3 | 13.1 | 13.0 | 1.03 |
| 9 | 18.2 | 21.7 | 21.1 | 18.9 | 20 | 19.9 | 1.34 |
| 10 | 27 | 29.3 | 28.9 | 27.5 | 28.1 | 28.1 | 0.87 |
| 11 | 35.3 | 40.6 | 39.6 | 36.3 | 37.8 | 37.9 | 2.04 |
| 12 | 51.1 | 53 | 52.6 | 51.5 | 52 | 52.0 | 0.73 |
| 13 | 59.2 | 67.6 | 66 | 60.8 | 63.4 | 63.4 | 3.21 |
| 14 | 65.1 | 71.2 | 70 | 66.3 | 68.1 | 68.1 | 2.36 |
| 15 | 71 | 78.1 | 76.7 | 72.3 | 74.6 | 74.5 | 2.74 |
| 16 | 83.3 | 86.4 | 85.8 | 83.9 | 84.9 | 84.8 | 1.21 |
| 17 | 87.8 | 91.4 | 90.7 | 88.5 | 89.7 | 89.6 | 1.36 |
| 18 | 88.8 | 92 | 91.4 | 89.4 | 90.5 | 90.4 | 1.25 |
| 19 | 91 | 94.1 | 93.5 | 91.6 | 92.5 | 92.5 | 1.20 |

2.3 The table shows the five sets of data and their means and standard deviations in Figure B（Inside）.

| Time（day） | data 1（cm） | data 2（cm） | data 3（cm） | data 4（cm） | data 5（cm） | average（cm） | standard deviation |
| --- | --- | --- | --- | --- | --- | --- | --- |
| 8 | 6.8 | 8 | 8.1 | 7.1 | 7.8 | 7.4 | 0.52 |
| 9 | 11.2 | 14 | 13.4 | 11.8 | 13 | 12.6 | 1.03 |
| 10 | 24.5 | 26.5 | 26.1 | 24.9 | 25.9 | 25.5 | 0.75 |
| 11 | 33.4 | 42.4 | 40.7 | 35.1 | 38.3 | 37.9 | 3.36 |
| 12 | 36.3 | 43.9 | 42.4 | 37.8 | 40.5 | 40.1 | 2.81 |
| 13 | 49.5 | 56.3 | 54.9 | 50.9 | 53.3 | 52.9 | 2.5 |
| 14 | 58.9 | 64.5 | 63.4 | 60 | 62.1 | 61.7 | 2.08 |
| 15 | 69 | 72.5 | 71.9 | 69.7 | 71.3 | 70.8 | 1.32 |
| 16 | 76.3 | 85.7 | 83.9 | 78.1 | 81.4 | 81.0 | 3.5 |
| 17 | 80.2 | 91.6 | 89.4 | 82.4 | 86.3 | 85.9 | 4.23 |
| 18 | 81.8 | 94.2 | 91.8 | 84.2 | 88.4 | 88.0 | 4.6 |
| 19 | 86.4 | 97 | 94.9 | 88.5 | 92.1 | 91.7 | 3.92 |

2.4 The table shows the five sets of data and their means and standard deviations in Figure B（Outside）.

| Time（day） | data 1（cm） | data 2（cm） | data 3（cm） | data 4（cm） | data 5（cm） | average（cm） | standard deviation |
| --- | --- | --- | --- | --- | --- | --- | --- |
| 8 | 4.8 | 6.8 | 6.4 | 5.2 | 6.2 | 5.8 | 0.75 |
| 9 | 10 | 12.4 | 11.9 | 10.5 | 11.6 | 11.2 | 0.89 |
| 10 | 14.9 | 17.5 | 17 | 15.4 | 16.6 | 16.2 | 0.98 |
| 11 | 23.2 | 29.6 | 28.4 | 24.4 | 26.8 | 26.4 | 2.39 |
| 12 | 35.8 | 39.8 | 39 | 36.6 | 38.2 | 37.8 | 1.48 |
| 13 | 45.4 | 55 | 53.1 | 47.3 | 50.6 | 50.2 | 3.55 |
| 14 | 55 | 64 | 62.3 | 56.7 | 59.9 | 59.5 | 3.36 |
| 15 | 63.9 | 70.9 | 69.5 | 65.3 | 67.8 | 67.4 | 2.59 |
| 16 | 78.3 | 85.5 | 84.1 | 79.7 | 82.3 | 81.9 | 2.67 |
| 17 | 88.9 | 92.5 | 91.8 | 89.6 | 91.1 | 90.7 | 1.34 |
| 18 | 92 | 97.2 | 96.2 | 93 | 95 | 94.6 | 1.94 |
| 19 | 98.4 | 101.6 | 100.9 | 99.1 | 100.4 | 100 | 1.17 |

2.5 The table shows the five sets of data and their means and standard deviations in Figure C（Inside）

| Time（day） | data 1（cm） | data 2（cm） | data 3（cm） | data 4（cm） | data 5（cm） | average（cm） | standard deviation |
| --- | --- | --- | --- | --- | --- | --- | --- |
| 12 | 13.3 | 16.3 | 15.7 | 13.9 | 15.2 | 14.8 | 1.12 |
| 13 | 18.5 | 21.7 | 21 | 19.2 | 20.5 | 20.1 | 1.17 |
| 14 | 23.5 | 28.3 | 27.4 | 24.4 | 26.3 | 25.9 | 1.8 |
| 15 | 29.5 | 35.9 | 34.7 | 30.7 | 33.1 | 32.7 | 2.39 |
| 16 | 35.1 | 42.7 | 41.2 | 36.6 | 39.3 | 38.9 | 2.81 |
| 17 | 51.3 | 60.7 | 58.8 | 53.2 | 56.4 | 56.0 | 3.46 |
| 18 | 64.1 | 71.7 | 70.2 | 65.6 | 68.3 | 67.9 | 2.81 |
| 19 | 71.2 | 84 | 81.5 | 73.7 | 78 | 77.6 | 4.74 |

2.6 The table shows the five sets of data and their means and standard deviations in Figure C（Outside）.

| Time（day） | data 1（cm） | data 2（cm） | data 3（cm） | data 4（cm） | data 5（cm） | average（cm） | standard deviation |
| --- | --- | --- | --- | --- | --- | --- | --- |
| 12 | 11.7 | 14.5 | 13.9 | 12.3 | 13.5 | 13.1 | 1.03 |
| 13 | 15.5 | 20.1 | 19.2 | 16.4 | 18.2 | 17.8 | 1.71 |
| 14 | 20.6 | 29.4 | 27.7 | 22.3 | 25.4 | 25.0 | 3.27 |
| 15 | 28.6 | 41.8 | 39.2 | 31.2 | 35.6 | 35.2 | 4.88 |
| 16 | 38.7 | 45.1 | 43.8 | 40 | 42.3 | 41.9 | 2.36 |
| 17 | 52.5 | 57.3 | 56.3 | 53.5 | 55.3 | 54.9 | 1.76 |
| 18 | 63.6 | 67.8 | 67 | 64.4 | 66.1 | 65.7 | 1.57 |
| 19 | 78.8 | 82.2 | 81.6 | 79.4 | 80.9 | 80.5 | 1.29 |

2.7 The table shows the five sets of data and their means and standard deviations in Figure D（Inside）

| Time（day） | data 1（cm） | data 2（cm） | data 3（cm） | data 4（cm） | data 5（cm） | average（cm） | standard deviation |
| --- | --- | --- | --- | --- | --- | --- | --- |
| 6 | 1.2 | 1.4 | 1.4 | 1.2 | 1.3 | 0.9 | 0.09 |
| 7 | 2.1 | 2.9 | 3.1 | 2.7 | 2.9 | 2.5 | 0.34 |
| 8 | 5.2 | 6.6 | 6.3 | 5.5 | 6.3 | 5.9 | 0.53 |
| 9 | 10.9 | 13.3 | 12.8 | 11.4 | 12.5 | 12.1 | 0.89 |
| 10 | 20.4 | 22.2 | 21.9 | 20.7 | 21.7 | 21.3 | 0.7 |
| 11 | 21.6 | 25 | 24.3 | 22.3 | 23.7 | 23.3 | 1.26 |
| 12 | 27.1 | 31.7 | 30.8 | 28 | 29.8 | 29.4 | 1.71 |
| 13 | 35.9 | 39.1 | 38.5 | 36.5 | 37.9 | 37.5 | 1.2 |
| 14 | 39.2 | 42.8 | 42.1 | 39.9 | 41.4 | 41.0 | 1.34 |
| 15 | 44.8 | 48.6 | 47.9 | 45.5 | 47.1 | 46.7 | 1.43 |
| 16 | 43.5 | 52.7 | 50.9 | 45.3 | 48.5 | 48.1 | 3.41 |
| 17 | 47.8 | 55.2 | 53.7 | 49.3 | 51.9 | 51.5 | 2.73 |
| 18 | 50.6 | 56.4 | 55.2 | 51.8 | 53.9 | 53.5 | 2.13 |
| 19 | 51.4 | 59.8 | 58.1 | 53.1 | 56 | 55.6 | 3.1 |

2.8 The table shows the five sets of data and their means and standard deviations in Figure D（Outside）.

| Time（day） | data 1（cm） | data 2（cm） | data 3（cm） | data 4（cm） | data 5（cm） | average（cm） | standard deviation |
| --- | --- | --- | --- | --- | --- | --- | --- |
| 7 | 2.4 | 3.8 | 3.5 | 2.7 | 3.5 | 3.1 | 0.53 |
| 8 | 5.3 | 7.1 | 6.7 | 5.7 | 6.6 | 6.2 | 0.67 |
| 9 | 8.6 | 10.8 | 10.4 | 9 | 10.1 | 9.7 | 0.84 |
| 10 | 12.6 | 14.2 | 13.9 | 12.9 | 13.8 | 13.4 | 0.62 |
| 11 | 21.1 | 24.9 | 24.2 | 21.8 | 23.4 | 23.0 | 1.43 |
| 12 | 31.1 | 34.5 | 33.8 | 31.8 | 33.2 | 32.8 | 1.26 |
| 13 | 42.3 | 45.7 | 45 | 43 | 44.4 | 44.0 | 1.26 |
| 14 | 41.1 | 50.1 | 48.3 | 42.9 | 46 | 45.6 | 3.32 |
| 15 | 44 | 50.6 | 49.3 | 45.3 | 47.7 | 47.3 | 2.45 |
| 16 | 50 | 54.4 | 53.5 | 50.9 | 52.6 | 52.2 | 1.62 |
| 17 | 51.2 | 55.4 | 54.6 | 52 | 53.7 | 53.3 | 1.57 |
| 18 | 50.3 | 59.1 | 57.4 | 52 | 55.1 | 54.7 | 3.27 |
| 19 | 54.1 | 58.7 | 57.8 | 55 | 56.8 | 56.4 | 1.71 |

2.9 The table shows the five sets of data and their means and standard deviations in Figure E（Inside）

| Time（day） | data 1（cm） | data 2（cm） | data 3（cm） | data 4（cm） | data 5（cm） | average（cm） | standard deviation |
| --- | --- | --- | --- | --- | --- | --- | --- |
| 8 | 1.9 | 2.9 | 2.8 | 2.4 | 2.6 | 2.2 | 0.35 |
| 9 | 4.8 | 6.8 | 6.4 | 5.2 | 6.2 | 5.8 | 0.75 |
| 10 | 11.7 | 13.3 | 13 | 12 | 12.9 | 12.5 | 0.62 |
| 11 | 18.3 | 24.3 | 23.1 | 19.5 | 21.7 | 21.3 | 2.22 |
| 12 | 20.8 | 27 | 25.8 | 22 | 24.3 | 23.9 | 2.31 |
| 13 | 31.1 | 36.7 | 35.6 | 32.2 | 34.3 | 33.9 | 2.08 |
| 14 | 37.2 | 42 | 41 | 38.2 | 40 | 39.6 | 1.76 |
| 15 | 43.3 | 48.3 | 47.3 | 44.3 | 46.2 | 45.8 | 1.85 |
| 16 | 45.9 | 53.3 | 51.8 | 47.4 | 50 | 49.6 | 2.73 |
| 17 | 53 | 63 | 61 | 55 | 58.4 | 58.0 | 3.69 |
| 18 | 54 | 65.4 | 63.1 | 56.3 | 60.1 | 59.7 | 4.2 |
| 19 | 59.4 | 70.8 | 68.6 | 61.6 | 65.5 | 65.1 | 4.23 |

2.10 The table shows the five sets of data and their means and standard deviations in Figure E（Outside）

| Time（day） | data 1（cm） | data 2（cm） | data 3（cm） | data 4（cm） | data 5（cm） | average（cm） | standard deviation |
| --- | --- | --- | --- | --- | --- | --- | --- |
| 8 | 2 | 2.2 | 2.5 | 2.1 | 2.3 | 1.9 | 0.17 |
| 9 | 3.7 | 5.7 | 5.3 | 4.1 | 5.1 | 4.7 | 0.75 |
| 10 | 8 | 10.4 | 9.9 | 8.5 | 9.6 | 9.2 | 0.89 |
| 11 | 13.5 | 16.1 | 15.6 | 14 | 15.2 | 14.8 | 0.98 |
| 12 | 24.2 | 30.4 | 29.2 | 25.4 | 27.7 | 27.3 | 2.31 |
| 13 | 33.6 | 44.6 | 42.5 | 35.7 | 39.5 | 39.1 | 4.09 |
| 14 | 42 | 49.2 | 47.8 | 43.4 | 46 | 45.6 | 2.67 |
| 15 | 54.2 | 58.8 | 57.9 | 55.1 | 56.9 | 56.5 | 1.71 |
| 16 | 56.2 | 66.8 | 64.7 | 58.3 | 61.9 | 61.5 | 3.92 |
| 17 | 63.3 | 66.1 | 65.6 | 63.8 | 65.1 | 64.7 | 1.06 |
| 18 | 64.8 | 67.8 | 67.2 | 65.4 | 66.7 | 66.3 | 1.12 |
| 19 | 64.7 | 74.9 | 72.9 | 66.7 | 70.2 | 69.8 | 3.78 |

2.11 The table shows the five sets of data and their means and standard deviations in Figure F（Inside）

| Time（day） | data 1（cm） | data 2（cm） | data 3（cm） | data 4（cm） | data 5（cm） | average（cm） | standard deviation |
| --- | --- | --- | --- | --- | --- | --- | --- |
| 12 | 3.8 | 5 | 5.4 | 4.6 | 5 | 4.4 | 0.54 |
| 13 | 5.9 | 8.3 | 7.9 | 6.3 | 7.7 | 7.1 | 0.94 |
| 14 | 9.1 | 12.1 | 11.5 | 9.7 | 11.2 | 10.6 | 1.13 |
| 15 | 15.5 | 20.3 | 19.4 | 16.4 | 18.5 | 17.9 | 1.81 |
| 16 | 18.4 | 21.2 | 20.7 | 18.9 | 20.4 | 19.8 | 1.08 |
| 17 | 31.1 | 35.9 | 35 | 32 | 34.1 | 33.5 | 1.81 |
| 18 | 38.2 | 44.2 | 43 | 39.4 | 41.8 | 41.2 | 2.23 |
| 19 | 44.3 | 48.1 | 47.4 | 45 | 46.8 | 46.2 | 1.44 |

2.12 The table shows the five sets of data and their means and standard deviations in Figure F（Outside）

| Time（day） | data 1（cm） | data 2（cm） | data 3（cm） | data 4（cm） | data 5（cm） | average（cm） | standard deviation |
| --- | --- | --- | --- | --- | --- | --- | --- |
| 12 | 5.4 | 6.2 | 6.1 | 5.5 | 5.8 | 5.2 | 0.32 |
| 13 | 5 | 7.2 | 6.8 | 5.4 | 6.7 | 6.1 | 0.86 |
| 14 | 8.2 | 12.6 | 11.7 | 9.1 | 11 | 10.4 | 1.63 |
| 15 | 12 | 18.4 | 17.2 | 13.2 | 15.8 | 15.2 | 2.4 |
| 16 | 18.3 | 24.1 | 23 | 19.4 | 21.8 | 21.2 | 2.17 |
| 17 | 29 | 36 | 34.6 | 30.4 | 33.1 | 32.5 | 2.59 |
| 18 | 43.5 | 47.9 | 47.1 | 44.3 | 45.7 | 45.7 | 1.65 |
| 19 | 49.9 | 53.9 | 52.8 | 50.6 | 51.1 | 51.7 | 1.32 |

**S1.3 Figure 8 Stem thickness (Fig. A) and plant height (Fig. B) of tomato seedlings grown by two nursery methods, plant factory and greenhouse**

3.1 The table shows the five sets of data and their means and standard deviations in Figure A（Inside）.

| Time（day） | data 1（cm） | data 2（cm） | data 3（cm） | data 4（cm） | data 5（cm） | average（cm） | standard deviation |
| --- | --- | --- | --- | --- | --- | --- | --- |
| 5 | 0.62 | 0.72 | 0.70 | 0.64 | 0.64 | 0.67 | 0.036 |
| 6 | 0.66 | 0.76 | 0.74 | 0.68 | 0.69 | 0.71 | 0.033 |
| 7 | 0.88 | 0.90 | 0.90 | 0.88 | 0.92 | 0.89 | 0.008 |
| 8 | 1.04 | 1.16 | 1.14 | 1.06 | 1.05 | 1.10 | 0.044 |
| 9 | 1.23 | 1.33 | 1.31 | 1.25 | 1.32 | 1.28 | 0.037 |
| 10 | 1.59 | 1.65 | 1.64 | 1.60 | 1.64 | 1.62 | 0.02 |
| 11 | 1.77 | 1.93 | 1.90 | 1.80 | 1.79 | 1.85 | 0.052 |
| 12 | 1.89 | 2.03 | 2.00 | 1.92 | 1.93 | 1.96 | 0.047 |
| 13 | 2.09 | 2.25 | 2.22 | 2.12 | 2.14 | 2.17 | 0.058 |
| 14 | 2.19 | 2.29 | 2.27 | 2.23 | 2.22 | 2.24 | 0.034 |
| 15 | 2.54 | 2.67 | 2.64 | 2.54 | 2.55 | 2.59 | 0.053 |
| 16 | 2.43 | 2.69 | 2.64 | 2.48 | 2.56 | 2.56 | 0.092 |
| 17 | 2.89 | 3.27 | 3.20 | 2.96 | 3.08 | 3.08 | 0.128 |
| 18 | 3.01 | 3.23 | 3.19 | 3.09 | 3.05 | 3.12 | 0.073 |
| 19 | 3.22 | 3.45 | 3.41 | 3.29 | 3.38 | 3.35 | 0.066 |

3.2 The table shows the five sets of data and their means and standard deviations in Figure A（Outside）.

| Time（day） | data 1（cm） | data 2（cm） | data 3（cm） | data 4（cm） | data 5（cm） | average（cm） | standard deviation |
| --- | --- | --- | --- | --- | --- | --- | --- |
| 7 | 0.75 | 0.83 | 0.85 | 0.77 | 0.84 | 0.81 | 0.046 |
| 8 | 0.91 | 1.04 | 1.03 | 0.97 | 1.05 | 1.00 | 0.032 |
| 9 | 1.05 | 1.11 | 1.13 | 1.06 | 1.04 | 1.08 | 0.023 |
| 10 | 1.25 | 1.32 | 1.30 | 1.22 | 1.23 | 1.26 | 0.046 |
| 11 | 1.64 | 1.74 | 1.69 | 1.65 | 1.63 | 1.67 | 0.023 |
| 12 | 1.81 | 1.87 | 1.86 | 1.82 | 1.84 | 1.84 | 0.023 |
| 13 | 1.83 | 2.13 | 2.07 | 1.93 | 2.05 | 2.00 | 0.082 |
| 14 | 2.13 | 2.15 | 2.18 | 2.14 | 2.12 | 2.16 | 0.023 |
| 15 | 2.57 | 2.67 | 2.61 | 2.59 | 2.67 | 2.62 | 0.037 |
| 16 | 2.70 | 2.88 | 2.83 | 2.73 | 2.73 | 2.78 | 0.06 |
| 17 | 3.05 | 3.16 | 3.13 | 3.03 | 3.02 | 3.08 | 0.06 |
| 18 | 3.02 | 3.16 | 3.18 | 3.06 | 3.17 | 3.12 | 0.074 |
| 19 | 3.17 | 3.27 | 3.25 | 3.19 | 3.22 | 3.22 | 0.037 |

3.3 The table shows the five sets of data and their means and standard deviations in Figure B（Inside）

| Time（day） | data 1（cm） | data 2（cm） | data 3（cm） | data 4（cm） | data 5（cm） | average（cm） | standard deviation |
| --- | --- | --- | --- | --- | --- | --- | --- |
| 5 | 2.9 | 3.3 | 3.2 | 3 | 3.1 | 2.5 | 0.14 |
| 6 | 2.9 | 3.7 | 3.5 | 3.1 | 3.3 | 2.7 | 0.28 |
| 7 | 3.2 | 3.6 | 3.5 | 3.3 | 3.4 | 2.8 | 0.14 |
| 8 | 3.3 | 3.9 | 3.8 | 3.4 | 3.6 | 3.0 | 0.23 |
| 9 | 3.6 | 4 | 3.9 | 3.7 | 3.8 | 3.2 | 0.14 |
| 10 | 4.8 | 5.8 | 5.6 | 5 | 5.3 | 4.7 | 0.37 |
| 11 | 5.3 | 5.9 | 5.8 | 5.4 | 5.6 | 5.0 | 0.23 |
| 12 | 5.8 | 6.4 | 6.3 | 5.9 | 6.1 | 5.5 | 0.23 |
| 13 | 6.5 | 7.1 | 7 | 6.6 | 6.8 | 6.2 | 0.23 |
| 14 | 6.7 | 7.9 | 8.3 | 7.5 | 7.9 | 7.3 | 0.54 |
| 15 | 9.1 | 10.3 | 10.6 | 10 | 10.3 | 9.7 | 0.52 |
| 16 | 12.7 | 16.5 | 15.7 | 13.5 | 15.2 | 14.6 | 1.41 |
| 17 | 14.5 | 17.9 | 17.2 | 15.2 | 16.8 | 16.2 | 1.27 |
| 18 | 16.8 | 18.8 | 18.4 | 17.2 | 18.4 | 17.8 | 0.78 |
| 19 | 17.3 | 20.7 | 20 | 18 | 19.6 | 19.0 | 1.27 |

3.4 The table shows the five sets of data and their means and standard deviations in Figure B（Outside）.

| Time（day） | data 1（cm） | data 2（cm） | data 3（cm） | data 4（cm） | data 5（cm） | average（cm） | standard deviation |
| --- | --- | --- | --- | --- | --- | --- | --- |
| 7 | 3.5 | 3.9 | 3.8 | 3.6 | 3.7 | 3.1 | 0.14 |
| 8 | 3.9 | 4.3 | 4.2 | 4 | 4.1 | 3.5 | 0.14 |
| 9 | 5 | 6 | 5.8 | 5.2 | 5.5 | 4.9 | 0.37 |
| 10 | 5.4 | 6 | 5.9 | 5.5 | 5.7 | 5.1 | 0.23 |
| 11 | 7 | 8 | 7.8 | 7.2 | 7.5 | 6.9 | 0.37 |
| 12 | 7.8 | 8.4 | 8.3 | 7.9 | 8.1 | 7.5 | 0.23 |
| 13 | 9.3 | 10.3 | 10.1 | 9.5 | 9.8 | 9.2 | 0.37 |
| 14 | 11.8 | 12.4 | 12.3 | 11.9 | 12.1 | 11.5 | 0.23 |
| 15 | 15.3 | 16.9 | 17.2 | 15.6 | 16.7 | 16.1 | 0.75 |
| 16 | 20.4 | 21 | 20.9 | 20.5 | 20.7 | 20.1 | 0.23 |
| 17 | 23.4 | 23.8 | 23.7 | 23.5 | 23.6 | 23.0 | 0.14 |
| 18 | 23.2 | 25.4 | 24.9 | 23.7 | 24.9 | 24.3 | 0.83 |
| 19 | 26.3 | 27.3 | 27.1 | 26.5 | 26.8 | 26.2 | 0.37 |

**S1.4 Figure 9 Comparison of different lateral branch lengths (Fig. A) and lateral branch widths (Fig. B) of tomato in the two nursery methods**

**4.1 The table shows the five sets of data and their means and standard deviations in Figure A**

| Time（day） | data 1（mm） | data 2（mm） | data 3（mm） | data 4（mm） | data 5（mm） | average（mm） | standard deviation |
| --- | --- | --- | --- | --- | --- | --- | --- |
| Inside lateral branch 1 | | | | | | | |
| 17 | 150.3 | 154.9 | 154 | 151.2 | 153.2 | 152.6 | 1.72 |
| 18 | 173 | 179.6 | 178.3 | 174.3 | 176.9 | 176.3 | 2.45 |
| 19 | 238.9 | 244.3 | 243.2 | 240 | 242.2 | 241.6 | 2.01 |
| 20 | 278.8 | 286.4 | 284.9 | 280.3 | 283.2 | 282.6 | 2.82 |
| 21 | 299.3 | 303.3 | 302.5 | 300.1 | 301.9 | 301.3 | 1.49 |
| Inside lateral branch 2 | | | | | | | |
| 17 | 142.8 | 147.8 | 146.8 | 143.8 | 145.9 | 145.3 | 1.86 |
| 18 | 171.1 | 175.5 | 174.6 | 172 | 173.9 | 173.3 | 1.63 |
| 19 | 222.5 | 228.7 | 227.5 | 223.7 | 226.2 | 225.6 | 2.31 |
| 20 | 250.8 | 256.4 | 255.3 | 251.9 | 254.2 | 253.6 | 2.09 |
| 21 | 272.9 | 280.3 | 278.9 | 274.3 | 277.2 | 276.6 | 2.77 |
| Inside lateral branch 3 | | | | | | | |
| 17 | 136.3 | 142.9 | 141.6 | 137.6 | 140.2 | 139.6 | 2.45 |
| 18 | 160.5 | 168.2 | 166.6 | 162 | 164.8 | 164.3 | 2.84 |
| 19 | 200 | 204 | 203.2 | 200.8 | 202.6 | 202.0 | 1.49 |
| 20 | 235.7 | 240.9 | 239.9 | 236.7 | 238.9 | 238.3 | 1.95 |
| 21 | 269.6 | 275 | 274 | 270.6 | 272.9 | 272.3 | 2.03 |
| Outside lateral branch1 | | | | | | | |
| 17 | 144.5 | 150.1 | 149 | 145.6 | 147.9 | 147.3 | 2.09 |
| 18 | 168.9 | 175.1 | 173.9 | 170.1 | 172.6 | 172.0 | 2.31 |
| 19 | 230.3 | 237.7 | 236.3 | 231.7 | 234.6 | 234.0 | 2.77 |
| 20 | 265.4 | 269.8 | 268.9 | 266.3 | 268.2 | 267.6 | 1.63 |
| 21 | 291.1 | 296.1 | 295.1 | 292.1 | 294.2 | 293.6 | 1.86 |
| Outside lateral branch 2 | | | | | | | |
| 17 | 141.2 | 146 | 145.1 | 142.1 | 144.2 | 143.6 | 1.81 |
| 18 | 172 | 178.6 | 177.3 | 173.3 | 175.9 | 175.3 | 2.45 |
| 19 | 196.6 | 203.4 | 202.1 | 197.9 | 200.6 | 200.0 | 2.54 |
| 20 | 248 | 252.6 | 251.7 | 248.9 | 250.9 | 250.3 | 1.72 |
| 21 | 280.7 | 285.9 | 284.9 | 281.7 | 283.9 | 283.3 | 1.95 |
| Outside lateral branch 3 | | | | | | | |
| 17 | 134.1 | 139.9 | 138.8 | 135.2 | 137.6 | 137.0 | 2.17 |
| 18 | 157.8 | 164.2 | 162.9 | 159.1 | 161.6 | 161.0 | 2.37 |
| 19 | 187.9 | 192.8 | 191.8 | 188.8 | 190.8 | 190.3 | 1.83 |
| 20 | 233.9 | 239.3 | 238.2 | 235 | 237.2 | 236.6 | 2 |
| 21 | 263.4 | 266.6 | 266 | 264 | 265.6 | 265.0 | 1.22 |

**4.2 The table shows the five sets of data and their means and standard deviations in Figure B**

| Time（day） | data 1（mm） | data 2（mm） | data 3（mm） | data 4（mm） | data 5（mm） | average（mm） | standard deviation |
| --- | --- | --- | --- | --- | --- | --- | --- |
| Inside lateral branch 1 | | | | | | | |
| 17 | 23.6 | 30.8 | 29.4 | 25 | 27.8 | 27.2 | 2.68 |
| 18 | 36 | 43.8 | 42.2 | 37.6 | 40.5 | 39.9 | 2.87 |
| 19 | 61.5 | 66.9 | 65.8 | 62.6 | 64.8 | 64.2 | 2 |
| 20 | 94.7 | 98.5 | 97.7 | 95.5 | 97.2 | 96.6 | 1.41 |
| 21 | 127.5 | 132.7 | 131.7 | 128.5 | 130.7 | 130.1 | 1.95 |
| Inside lateral branch 2 | | | | | | | |
| 17 | 32.1 | 36.9 | 36 | 33 | 35.1 | 34.5 | 1.81 |
| 18 | 53.4 | 57.8 | 56.9 | 54.3 | 56.2 | 55.6 | 1.63 |
| 19 | 80.6 | 87.4 | 86.1 | 81.9 | 84.6 | 84.0 | 2.54 |
| 20 | 120.4 | 125.6 | 124.6 | 121.4 | 123.6 | 123.0 | 1.95 |
| 21 | 145.1 | 151.7 | 150.4 | 146.4 | 149 | 148.4 | 2.45 |
| Inside lateral branch 3 | | | | | | | |
| 17 | 25.6 | 32.2 | 30.9 | 26.9 | 29.5 | 28.9 | 2.45 |
| 18 | 42 | 49.2 | 47.8 | 43.4 | 46.2 | 45.6 | 2.68 |
| 19 | 71 | 75 | 74.2 | 71.8 | 73.6 | 73.0 | 1.49 |
| 20 | 108.4 | 113.6 | 112.6 | 109.4 | 111.6 | 111.0 | 1.95 |
| 21 | 131.2 | 136 | 135.1 | 132.1 | 134.2 | 133.6 | 1.81 |
| Outside lateral branch1 | | | | | | | |
| 17 | 23.5 | 28.5 | 27.5 | 24.5 | 26.6 | 26.0 | 1.86 |
| 18 | 41.6 | 47.6 | 46.4 | 42.8 | 45.2 | 44.6 | 2.23 |
| 19 | 61.5 | 66.9 | 65.9 | 62.5 | 64.8 | 64.2 | 2.03 |
| 20 | 85.7 | 93.1 | 91.7 | 87.1 | 90 | 89.4 | 2.77 |
| 21 | 116.2 | 120.6 | 119.7 | 117.1 | 119 | 118.4 | 1.63 |
| Outside lateral branch 2 | | | | | | | |
| 17 | 38.7 | 43.3 | 42.4 | 39.6 | 41.6 | 41.0 | 1.72 |
| 18 | 54.6 | 59.4 | 58.5 | 55.5 | 57.6 | 57.0 | 1.81 |
| 19 | 76.8 | 83.6 | 82.3 | 78.1 | 80.8 | 80.2 | 2.54 |
| 20 | 117.5 | 122.9 | 121.8 | 118.6 | 120.8 | 120.2 | 2 |
| 21 | 133.7 | 140.7 | 139.3 | 135.1 | 137.8 | 137.2 | 2.59 |
| Outside lateral branch 3 | | | | | | | |
| 17 | 17.5 | 21.1 | 20.2 | 18.4 | 19.7 | 19.3 | 1.82 |
| 18 | 45.9 | 49.7 | 48.8 | 46.8 | 48.2 | 47.8 | 1.91 |
| 19 | 67.9 | 72.5 | 71.4 | 69 | 70.6 | 70.2 | 2.34 |
| 20 | 102 | 104.8 | 104.1 | 102.7 | 103.8 | 103.4 | 1.42 |
| 21 | 130.1 | 135.1 | 133.8 | 131.4 | 133 | 132.6 | 2.53 |

**S1..5 Figure 10 Comparison of stem thickness at true leaf stage between the two nursery methods**

| Time（day） | data 1（cm） | data 2（cm） | data 3（cm） | data 4（cm） | data 5（cm） | average（cm） | standard deviation |
| --- | --- | --- | --- | --- | --- | --- | --- |
| Stem thickness at true leaf stage(Inside) | | | | | | | |
| 7 | 4.4 | 5.0 | 4.9 | 4.5 | 4.7 | 4.3 | 0.19 |
| 8 | 4.7 | 5.1 | 5 | 4.8 | 4.9 | 4.5 | 0.15 |
| 9 | 5.2 | 5.6 | 5.5 | 5.3 | 5.4 | 5.0 | 0.16 |
| 10 | 5.6 | 6.0 | 5.9 | 5.7 | 5.8 | 5.4 | 0.15 |
| 11 | 6.0 | 6.2 | 6.2 | 6.0 | 6.1 | 5.7 | 0.11 |
| 12 | 6.2 | 6.8 | 6.7 | 6.3 | 6.5 | 6.1 | 0.19 |
| 13 | 6 | 7.6 | 7.3 | 6.3 | 7.2 | 6.8 | 0.55 |
| 14 | 6.4 | 7.6 | 7.4 | 6.6 | 7.4 | 7.0 | 0.44 |
| 15 | 6.4 | 7.6 | 7.3 | 6.7 | 7.4 | 7.0 | 0.39 |
| 16 | 6.8 | 7.8 | 7.6 | 7.0 | 7.7 | 7.3 | 0.39 |
| 17 | 6.8 | 7.8 | 7.6 | 7.0 | 7.7 | 7.3 | 0.39 |
| 18 | 6.9 | 8.1 | 7.9 | 7.1 | 7.9 | 7.5 | 0.42 |
| 19 | 7.0 | 8.4 | 8.1 | 7.3 | 8.1 | 7.7 | 0.47 |
| 20 | 7.0 | 9.4 | 8.9 | 7.5 | 8.6 | 8.2 | 0.86 |
| 21 | 7.4 | 9.0 | 8.7 | 7.7 | 8.6 | 8.2 | 0.59 |
| Stem thickness at true leaf stage(Outside) | | | | | | | |
| 7 | 3.2 | 4.0 | 4.2 | 3.8 | 4.0 | 3.6 | 0.34 |
| 8 | 3.9 | 4.9 | 4.8 | 4.4 | 4.6 | 4.2 | 0.35 |
| 9 | 3.8 | 4.8 | 5.0 | 4.4 | 4.7 | 4.3 | 0.42 |
| 10 | 5.2 | 5.4 | 5.7 | 5.3 | 5.5 | 5.1 | 0.17 |
| 11 | 4.9 | 5.7 | 5.9 | 5.5 | 5.7 | 5.3 | 0.34 |
| 12 | 5.6 | 6.2 | 6.1 | 5.7 | 5.9 | 5.5 | 0.23 |
| 13 | 5.2 | 6.2 | 6.4 | 5.8 | 6.1 | 5.7 | 0.42 |
| 14 | 5.6 | 6.6 | 6.4 | 6.2 | 6.5 | 6.1 | 0.36 |
| 15 | 5.9 | 6.7 | 6.9 | 6.5 | 6.7 | 6.3 | 0.34 |
| 16 | 6 | 6.9 | 6.8 | 6.6 | 6.9 | 6.48 | 0.34 |
| 17 | 6.2 | 7.1 | 7.3 | 6.8 | 7 | 6.65 | 0.38 |
| 18 | 6.3 | 7 | 7.3 | 6.8 | 6.9 | 6.63 | 0.33 |
| 19 | 6.1 | 7.6 | 7.3 | 6.4 | 7.4 | 6.88 | 0.60 |
| 20 | 6.1 | 8.2 | 7.8 | 6.5 | 7.4 | 7.12 | 0.79 |
| 21 | 7 | 8.4 | 8.1 | 7.2 | 8.1 | 7.67 | 0.55 |

**S1.6 Figure 11 Regression analysis of cumulative leaf length and width with days of grafting**

| Grafting days | Cumulative leaf length | Cumulative leaf width |
| --- | --- | --- |
| 7 | 2254.3 | 1447.7 |
| 8 | 2381.1 | 1539.3 |
| 9 | 2787.35 | 1788.4 |
| 10 | 3284.8 | 2119.1 |
| 11 | 3938.3 | 2574.4 |
| 12 | 4582.9 | 3003.65 |
| 13 | 5448.35 | 3641.45 |
| 14 | 6218.34 | 4090.5 |
| 15 | 6707.8 | 4519.4 |
| 16 | 7683.1 | 5320.22 |
| 17 | 8700.1 | 6051.65 |
| 18 | 9635.4 | 6860.7 |
| 19 | 10602.6 | 7733.1 |
| 20 | 11921.1 | 8807.85 |
| 21 | 12794.3 | 9462.3 |

| Grafting days | Cumulative leaf length | Cumulative leaf width |
| --- | --- | --- |
| 7 | 2011.6 | 1362.3 |
| 11 | 3725.15 | 2462.75 |
| 15 | 6806.875 | 4505.625 |
| 19 | 10610 | 7812.5 |

**S1.7 Figure 12 Statistics on the number of tomato seedling deaths in the two nursery methods**

| **Seedling tray number** | **Number of dead seedlings IN** | **Number of dead seedlings OUT** |
| --- | --- | --- |
| 1 | 4 | 4 |
| 2 | 6 | 6 |
| 3 | 3 | 3 |
| 4 | 6 | 6 |
| 5 | 8 | 8 |
| 6 | 5 | 5 |
| 7 | 3 | 3 |
| 8 | 6 | 6 |
| 9 | 4 | 4 |
| 10 | 5 | 5 |
| 11 | 6 | 6 |
| 12 | 2 | 2 |
| 13 | 3 | 3 |
| 14 | 5 | 5 |
| 15 | 6 | 6 |
| 16 | 4 | 4 |
| 17 | 6 | 6 |
| 18 | 7 | 7 |
| 19 | 9 | 9 |
| 20 | 5 | 5 |

**S1.8 Figure 13 Schematic diagram of electricity consumption during seedling rearing for the two seedling methods**

| **cultivation method** | **Cumulative electricity consumption(kwh)** |
| --- | --- |
| In8 | 20099 |
| Out8 | 64628 |
| In9 | 22511 |
| Out9 | 82760 |

**S1.9 Figure 14 Schematic diagram of fruit diameter for both nursery methods**

Data on the right

| Time（day） | data 1（cm） | data 2（cm） | data 3（cm） | data 4（cm） | data 5（cm） | average（cm） | standard deviation |
| --- | --- | --- | --- | --- | --- | --- | --- |
| lateral branch | | | | | | | |
| 7 | 26.9 | 30.7 | 30.0 | 27.6 | 29.2 | 28.8 | 1.36 |
| 14 | 29.5 | 34.7 | 33.7 | 30.5 | 32.5 | 32.1 | 1.86 |
| 21 | 32.7 | 34.5 | 34.2 | 33.0 | 34.0 | 33.6 | 0.66 |
| 28 | 32.8 | 35.6 | 35.1 | 33.3 | 34.6 | 34.2 | 1.02 |
| dichotomous branch | | | | | | | |
| 7 | 26.3 | 29.7 | 29.0 | 27.0 | 28.4 | 28.0 | 1.26 |
| 14 | 29.5 | 31.7 | 31.3 | 29.9 | 31.0 | 30.6 | 0.84 |
| 21 | 30.0 | 35.2 | 34.2 | 31.0 | 33.0 | 32.6 | 1.94 |
| 28 | 32.9 | 34.3 | 34.0 | 33.2 | 34.0 | 33.6 | 0.53 |
| trilobal branch | | | | | | | |
| 7 | 23.7 | 26.7 | 26.1 | 24.3 | 25.6 | 25.2 | 1.12 |
| 14 | 27.7 | 29.7 | 29.3 | 28.1 | 29.1 | 28.7 | 0.75 |
| 21 | 31 | 31.8 | 32 | 31.6 | 31.8 | 31.4 | 0.34 |
| 28 | 33.1 | 34.5 | 34.2 | 33.4 | 34.2 | 33.8 | 0.53 |

Data on the left

| Time（day） | data 1（cm） | data 2（cm） | data 3（cm） | data 4（cm） | data 5（cm） | average（cm） | standard deviation |
| --- | --- | --- | --- | --- | --- | --- | --- |
| lateral branch | | | | | | | |
| 7 | 27.6 | 29.2 | 28.9 | 27.9 | 28.7 | 28.4 | 0.62 |
| 14 | 29.3 | 34.9 | 33.8 | 30.4 | 32.3 | 32.1 | 2.08 |
| 21 | 31.8 | 34.8 | 34.2 | 32.4 | 33.7 | 33.3 | 1.12 |
| 28 | 33.5 | 35.3 | 34.9 | 33.9 | 34.8 | 34.4 | 0.67 |
| dichotomous branch | | | | | | | |
| 7 | 27.3 | 28.3 | 28.1 | 27.5 | 28.2 | 27.8 | 0.4 |
| 14 | 28.4 | 30.8 | 30.3 | 28.9 | 30 | 29.6 | 0.89 |
| 21 | 32.3 | 33.3 | 33.2 | 32.8 | 33 | 32.6 | 0.35 |
| 28 | 32.9 | 34.3 | 34 | 33.2 | 34 | 33.6 | 0.53 |
| trilobal branch | | | | | | | |
| 7 | 23.3 | 27.7 | 26.8 | 24.2 | 25.9 | 25.5 | 1.62 |
| 14 | 27.7 | 29.7 | 29.3 | 28.1 | 29.1 | 28.7 | 0.75 |
| 21 | 31 | 31.8 | 32 | 31.6 | 31.8 | 31.4 | 0.34 |
| 28 | 32.8 | 34.2 | 33.9 | 33.1 | 33.9 | 33.5 | 0.53 |
